# The C-reactive protein/albumin ratio predicts postoperative delirium in patients older than 60 years following total knee arthroplasty

**DOI:** 10.3389/fsurg.2022.814345

**Published:** 2022-08-16

**Authors:** Lin Zhang, Baoquan Li, Yujiang Bai, Xiaoshuang Liu, Xin Chai

**Affiliations:** ^1^ Orthopedic Surgery, The Second Affiliated Hospital of Qiqihar Medical University, Qiqihar, China; ^2^ Orthopedic Surgery, The Third Affiliated Hospital of Qiqihar Medical University, Qiqihar, China; ^3^ Endoscope Center, The Third Affiliated Hospital of Qiqihar Medical University, Qiqihar, China

**Keywords:** delirium, CRP, Alb, TKA, biomarker

## Abstract

**Purpose:**

This study aimed to investigate the relationship between serum C-reactive protein (CRP)/Albumin ratio (CAR) and postoperative delirium (POD) in patients older than 60 years following total knee arthroplasty (TKA).

**Methods:**

From January 2019 to January 2021, 268 patients were recruited in this prospective observational investigation. Patients with serum CRP, Alb, CAR, delirious status and delirious score were assessed. The effect of CRP, Alb, CAR on predicting delirium was assessed with the area under the receiver operating characteristic (ROC) curve (AUC).

**Results:**

The study found that higher CRP level (*P* < 0.001), low Alb level (*P* < 0.001), and higher CAR (*P* < 0.001) were independently associated with POD. The AUC of CAR for POD was 0.782, with the cut-off value of 0.117, a sensitivity of 83.3% and a specificity of 65.9% respectively (*P* < 0.001), suggesting that CAR had moderate efficacy on predicting POD occurrence than CRP (AUC: 0.761) and Alb (AUC: 0.300). The results also showed that age, ASA and the operation time was an independent predictor for patients with POD.

**Conclusions:**

Our findings demonstrated CAR may be an effective biomarker to predict postoperative delirium in patients over 60 years of age with TKA, which provides potential recommendations for early intervention in delirium care.

## Introduction

Total knee arthroplasty (TKA) can successfully relieve knee pain, restore function and improve quality of life (1). However, the majority of TKA patients are older than 60 years, and these patients are also associated with a number of comorbidities, which increases the likelihood of perioperative complications (2, 3). Postoperative delirium (POD) has become a major postoperative complication because it is closely associated with decreased quality of life, longer hospital stays, and increased morbidity and mortality (4, 5).

POD is a severe neuropsychiatric syndrome characterized by an acute deterioration of attention, cognition, and mental function (6). Previous studies have reported that the incidence of POD in adults with non-cardiovascular diseases ranges from 4% to 65%, whereas 35% to 65% of patients undergoing elective orthopedic surgery account for 9% to 15% (7). There is increasing evidence that POD may lead to permanent cognitive decline and dementia in some patients (8, 9). Most nurses are able to detect postoperative cognitive changes in patients, but early diagnosis and prevention of POD was critical to avoid compromising patient care (10). It is widely believed that surgery, especially major surgery, is associated with severe systemic inflammation (11). Since the brain responses to inflammation, including activation of apoptosis of microglia and neurons, can lead to synaptic and neurochemical disorders (12).

Current evidence has also shown that POD has been shown to be associated with inflammation of the central nervous system (13, 14). C-reactive protein (CRP), one of the most common markers of systemic inflammation, has been recognized as an independent risk factor for delirium after vascular surgery (15) and hip surgery (16). In addition, albumin (Alb), as a protein involved in acute inflammatory response, is often used to assess the nutritional status of surgical patients (17). At the same time, some studies have suggested that **hypoalbuminemia** is an important reason for the increased complications and reoperation rate in elderly patients with hip fracture (18). The ratio of C-reactive protein to albumin, named as CRP/Alb ratio (CAR), has been widely used as an indicator to predict the prognosis of cardiovascular events (19), liver surgery (20) and renal cancer surgery (21).

Therefore, the increased CAR level during perioperative period may be associated with the risk of POD in patients undergoing surgery. To test this hypothesis, we conducted a hospital-based study to investigate the relationship between perioperative CAR and the risk of POD in patients with TKA older than 60 years.

## Methods

### Study design

A prospective observational investigation was conducted from January 2018 to January 2021. The inclusion and exclusion criteria are as follows: (1) the patient underwent total knee replacement due to severe osteoarthritis; (2) the patient was older than 60 years; (3) the American Society of Anesthesiologists (ASA) scores does not exceed Grade III; (4) no history of central nervous damage or psychiatric medication in the recent 3 months; (5) no history of neuropsychiatric disease or dementia, no cognitive impairment; (6) no infectious diseases affecting CRP and Alb; (7) no immune diseases or severe liver and kidney disease. In this study, we used the Chinese Mini-Mental State Examination (MMSE) to assess the cognitive function for patients, which included 19 independent item scores totaling 30 (22), and patients with a MMSE score of less than 27 were excluded from the study if they were considered to have cognitive impairment.

### Clinical and laboratory data

The clinical and laboratory data for each patient including age, sex, ASA scores, medical disease, hemoglobin, platelets and white blood cells, lymphocyte, body mass index (BMI), CRP, Alb, CAR, the neutrophil to lymphocyte ratio (NLR), the platelet to lymphocyte ratio (PLR) was carefully recorded. The blood indicators were collected as follows: after admission, the patients' fasting external venous blood samples were taken preoperatively, and all the samples were sent to the laboratory for examination in our hospital within 2 h. In this study, the CAR was defined as the ratio of preoperative CRP to preoperative Alb.

### Clinical assessment

All patients were treated with TKA under general anesthesia by the same senior physician. Nonsteroidal drugs were used for perioperative analgesia. An experienced psychiatrist and nurse diagnosed postoperative patients with POD using the confusion assessment method (CAM, a qualitative measure of delirium state), and the memorial delirium assessment scale (MADS, a quantitative measure of delirium severity) (23). The main contents of CAM included four: (1) Acute onset and mood swings; (2) Inattention; (3) Thinking disorder; (4) Changes in consciousness level. If the patient has both (1) and (2), plus either (3) or (4), the diagnosis is made. In this study, we assessed the occurrence of POD within 5 days after surgery, and if POD occurred at least once within 5 days after surgery, they were defined as POD group.

### Statistical analyses

The categorical data is analyzed using the chi-square tests, and the Mann–Whitney *U*-tests was used to compare the differences in continuous data between the two independent groups. According to the coordinate points of the receiver operating characteristic (ROC) curve, the optimal threshold of CAR for POD was calculated by using the Youden index (*J*-statistic = sensitivity + specificity − 1). To clarify the results of the ROC curve, the test has “low accuracy” if the area under treatment (AUC) ranges from 0.5 to 0.7, it has a “mild accuracy” if the AUC ranges between 0.7 and 0.9, and it has a “high accuracy” if the AUC > is 0.9. To screen the potential risk factors for the pods, a multivariate logistic regression analysis was performed, and only those factors with *P* value < 0.1 were included in the final multivariate logistic regression model. The *P* value of <0.05 is considered statistically significant. In addition, regressions were adjusted for gender, preoperative comorbidities (such as diabetes mellitus, hypertension, coronary artery disease, peripheral vascular disease and central nervous system disease), etc. All statistical analyses were performed using SPSS Statistics version 21 (IBM Corp., Armonk, NY).

## Results

### Basic characteristics

**Figure 1:**
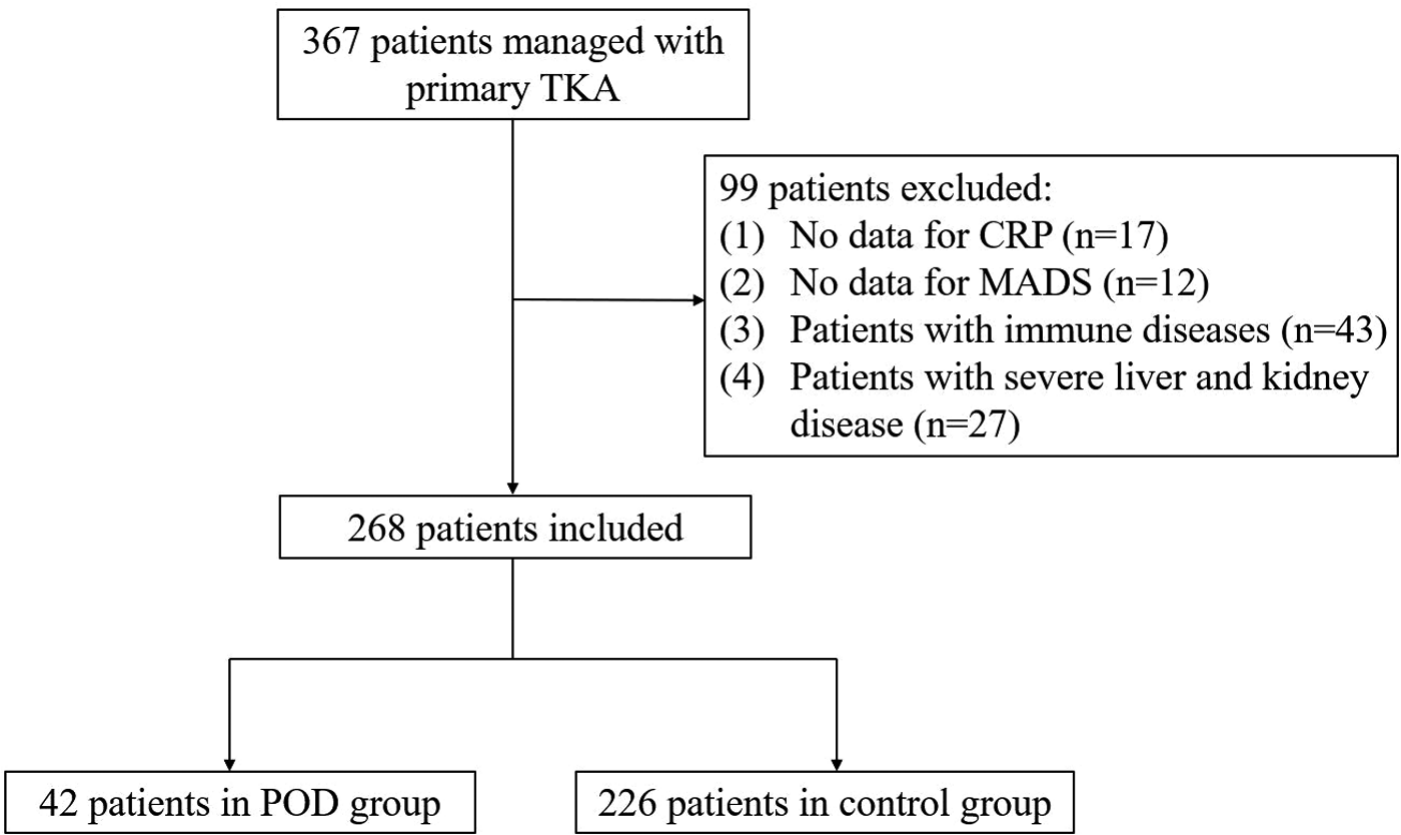
Flowchart of included and excluded cases. Aberrative: TKA, total knee arthroplasty; POD, postoperative delirium; MADS, memorial delirium assessment scale; CRP, C-reactive protein.

Following exclusion criteria, 367 patients were enrolled in our study (Figure 1), of which, 99 patients were excluded. Therefore, a total of 268 patients were finally included, of which the patients in POD group included 14 males and 28 females, with the mean age of 70.29 ± 5.03 years, while the Control group (without POD) included 80 males and 146 females, with the mean age of 66.56 ± 5.87 years, and it is was significant differences between the two groups (*P* = 0.001). The demographic characteristics and hematologic parameters for the patients and control groups were presented in Table 1.

**Table 1:** Demographic and clinical parameters of the POD and control group.

Characteristic	POD group (*n* = 42)	Control group (*n* = 226)	*P*-Value
Age (years)	70.29 ± 5.03	66.56 ± 5.87	0.001
Male/female (*n*)	14/28	80/146	0.796
BMI (kg/m^2^)	24.27 ± 3.18	24.04 ± 3.29	0.683
**Preoperative comorbidities, *n***	** **	** **	** **
**Diabetes mellitus**	**13**	**24**	**0**.**001**
**Hypertension**	**11**	**23**	**0**.**001**
**Coronary artery disease**	**6**	**14**	**0**.**067**
**Peripheral vascular disease**	**4**	**17**	**0**.**658**
**Central nervous system disease**	**2**	**6**	**0**.**461**
ASA (scores)	2.50 ± 0.59	2.21 ± 0.54	0.002
Preoperative MADS (scores)	0.36 ± 0.49	0.35 ± 0.48	0.926
Postoperative MADS (scores)	7.57 ± 0.98	1.01 ± 0.67	0.001
Hemoglobin (g/L)	127.71 ± 14.66	129.04 ± 18.12	0.655
Platelets × 10^9^/L	189.23 ± 51.85	189.10 ± 57.03	0.989
White blood cells × 10^9^/L	4.12 ± 1.48	4.24 ± 1.86	0.698
Lymphocyte × 10^9^/L	1.77 ± 0.49	1.70 ± 0.55	0.459
CRP (mg/L)	20.33 ± 32.75	8.00 ± 17.68	0.001
Alb (g/L)	39.16 ± 6.99	43.17 ± 4.13	0.001
CAR	0.54 ± 0.91	0.18 ± 0.40	0.001
NLR	2.54 ± 1.17	2.96 ± 2.63	0.313
PLR	117.36 ± 55.31	131.03 ± 104.10	0.408
The operation time (min)	138.23 ± 6.14	121.34 ± 5.69	0.001

**Aberrative:** POD, postoperative delirium; BMI, body mass index; ASA, American Society of Anesthesiologists; MADS, memorial delirium assessment scale; CRP, C-reactive protein; Alb, albumin; CAR, C-reactive protein to albumin Ratio; NLR, neutrophil-lymphocyte ratio; PLR, platelet-lymphocyte ratio; *n*, number; min, minutes. *P* < 0.05 was considered statistically significant.

### Laboratory measurements

The preoperative CRP, Alb and CAR of the patients in POD group were 20.33 ± 32.75 mg/L, 39.16 ± 6.99 g/L and 0.54 ± 0.91 respectively, and the patients in Control group had a CRP of 8.00 ± 17.68 mg/L, an Alb of 43.17 ± 4.13 g/L, and a CAR of 0.18 ± 0.40. There was a statistically significant difference in CRP, Alb and CAR between patients and controls (all of *P* < 0.001) (Table 1). The mean value of the MADS score after surgery were 6.20 ± 3.16 and 0.08 ± 0. 39 between the POD and Control groups (*P* < 0.001). However, the levels of hemoglobin, white blood cells, platelets and lymphocyte were no significantly in the POD patients than those in the control group (all of *P* > 0.05).

### CAR, ASA scores were associated with POD

**Figure 2:**
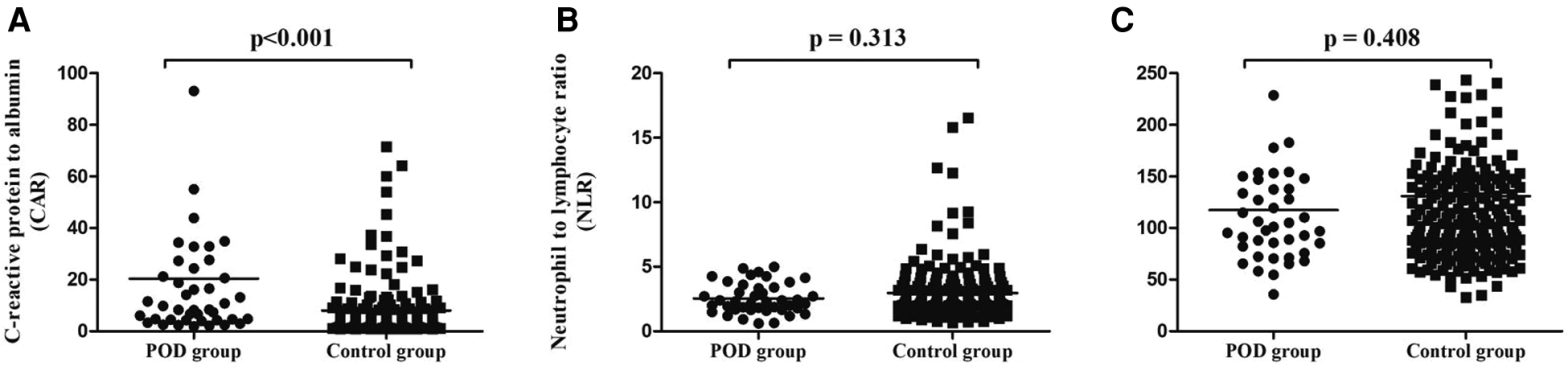
Comparison of CAR (**A**), NLR (**B**) and PLR (**C**) levels between POD and control groups. Aberrative: POD, postoperative delirium; CAR, C-reactive protein to albumin Ratio; NLR, neutrophil-lymphocyte ratio; PLR, platelet-lymphocyte ratio. *P* < 0.05 was considered statistically significant.

In this study, we assessed whether age, ASA scores, CRP, Alb, CAR, hemoglobin, platelets and white blood cells, lymphocyte, NLR, PLR and the operation time correlated with POD. As shown in Table 1 and Figure 2, there was a statistically significant difference in CAR (*P* < 0.001), but it was not significant difference in NLR (*P* = 0.313) and PLR (*P* = 0.408) between POD and Control groups. As shown in Table 2, we found that age, ASA scores, CRP, Alb, CRP and the operation time were positively correlated with the MADS postoperative score in this study (*r* = 0.235, *P* < 0.001; *r* = 0.138, *P* < 0.001; *r* = 0.164, *P* < 0.007; *r* = −0.252, *P* < 0.001; *r* = 0.190, *P* = 0.002, and *r* = 0.710, *P* = 0.001, respectively). Compared with hemoglobin, platelets, white blood cells, NLR and PLR, CAR was more strongly related to MADS by the Spearman correlation test (Table 2).

**Table 2:** Correlations of clinical parameters and CAR, NLR and PLR with laboratory measurements in POD patients.

Variable	MADS	
	Person *r*	*P* value
Age (years)	0.235	0.001
BMI (kg/m^2^)	0.019	0.758
ASA (scores)	0.138	0.024
Hemoglobin (g/L)	−0.035	0.572
Platelets × 10^9^/L	−0.015	0.806
White blood cells × 10^9^/L	−0.013	0.828
Lymphocyte × 10^9^/L	0.047	0.442
CRP (mg/L)	0.164	0.007
Alb (g/L)	−0.252	0.001
CAR	0.190	0.002
NLR	−0.062	0.313
PLR	−0.062	0.314
The operation time (min)	0.710	0.001

**Aberrative:** POD, postoperative delirium; BMI, body mass index; ASA, American Society of Anesthesiologists; CRP, C-reactive protein; Alb, albumin; CAR, C-reactive protein to albumin Ratio; NLR, neutrophil-lymphocyte ratio; PLR, platelet-lymphocyte ratio; min, minutes. *P* < 0.05 was considered statistically significant.

### Predictive value of CAR for POD

**Figure 3:**
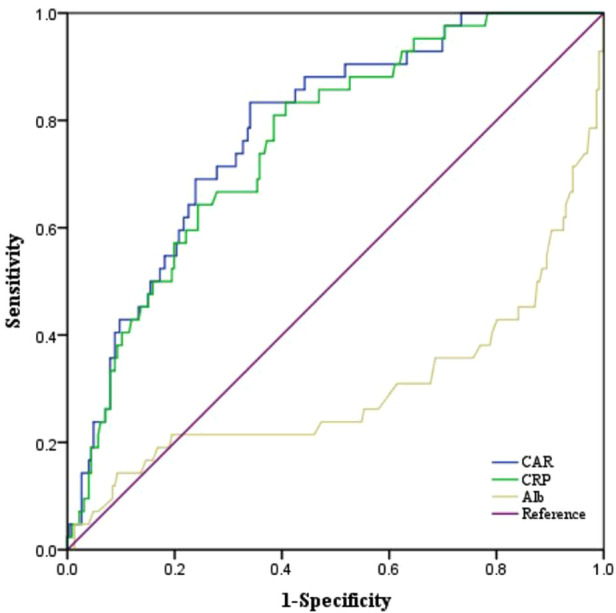
The ROC curves of CAR, CRP and Alb. Aberrative: ROC, receiver operating characteristic; CRP, C-reactive protein; Alb, albumin; CAR, C-reactive protein to albumin Ratio.

The predictive value of preoperative CAR for POD was evaluated by ROC curve analysis. As shown in Table 3 and Figure 3, the area under the curve (AUC) of CAR for POD was 0.782, with the cut-off value of 0.117, a sensitivity of 83.3% and a specificity of 65.9% respectively (*P* < 0.001), and the positive (+LR) and negative (−LR) likelihood ratio were 2.446 and 0.253, respectively, which of the AUC value is better than that of CRP (AUC: 0.761) and Alb (AUC: 0.300). Based on the ROC curve analysis, the results showed that the CAR has a moderate prediction effect on the occurrence of postoperative insanity after TKA over 60 years.

**Table 3:** Comparison of the AUC for the three inflammation-based prognostic scores.

Variable	AUC (95%CI)	Youden's Index	Predictive Cutoff	Sensitivity (%)	Specificity (%)	PPV (%)	NPV (%)	*P* value
CRP (mg/L)	0.761 (0.688–0.833)	0.336	9.29	50%	84.6%	40.5%	90.2%	0.001
Alb (mg/L)	0.300 (0.193–0.407)	0.399	40.75	35.7%	24.3%	8.1%	67.0%	0.001
CAR	0.782 (0.712–0.851)	0.493	0.117	83.3%	65.9%	31.2%	95.5%	0.001

**Aberrative:** POD, postoperative delirium; CRP, C-reactive protein; Alb, albumin; CAR, C-reactive protein to albumin Ratio; PPV, positive predictive value; NPV, negative predictive value. *P* < 0.05 was considered statistically significant.

### Logistic regression analysis of factors independently associated with POD

In addition, we performed logistic regression analysis to reveal the association of age, ASA scores, CRP, Alb, CAR, hemoglobin, platelets and white blood cells, lymphocyte, NLR and PLR in POD. Results showed that age, diabetes mellitus, hypertension, ASA scores, CRP, Alb, CAR and the operation time was an independent predictor for patients with POD (Table 4). After adjustment, the central nervous system disease and the operation time was an independent predictor for patients with POD (Table 5).

**Table 4:** Logistic regression analysis for POD patients (unadjusted).

Risk factor	OR (95% CI)	*P* value
Age (years)	1.116 (1.053–1.183)	<0.001
Female /Male	0.913 (0.454–1.832)	0.797
BMI (kg/m^2^)	1.021 (0.923–1.130)	0.682
ASA (scores)	2.559 (1.395–4.694)	0.002
Preoperative comorbidities		
Diabetes mellitus	3.773 (1.731–8.223)	0.001
Hypertension	3.132 (1.391–7.053)	0.006
Coronary artery disease	2.524 (0.910–6.996)	0.075
Peripheral vascular disease	1.774 (0.612–5.136)	0.291
Central nervous system disease	1.883 (0.357–9.408)	0.468
Hemoglobin (g/L)	0.996 (0.977–1.014)	0.654
Platelets × 10^9^/L	1.000 (0.994–1.006)	0.989
White blood cells × 10^9^/L	0.963 (0.795–1.166)	0.697
Lymphocyte × 10^9^/L	1.254 (0.690–2.280)	0.458
CRP (mg/L)	1.020 (1.005–1.034)	0.008
Alb (g/L)	0.848 (0.789–0.911)	<0.001
CAR	2.594 (1.336–5.035)	0.005
NLR	0.900 (0.733–1.105)	0.314
PLR	0.998 (0.993–1.003)	0.408
The operation time (min)	1.721 (1.404–2.110)	<0.001

**Aberrative:** POD, postoperative delirium; OR, Odds ratio; CI, Confidence interval; CRP, C-reactive protein; Alb, albumin; CAR, C-reactive protein to albumin Ratio; NLR, neutrophil-lymphocyte ratio; PLR, platelet-lymphocyte ratio; min, minutes. *P* < 0.05 was considered statistically significant.

**Table 5:** Logistic regression analysis for POD patients (adjusted).

Risk factor	OR (95% CI)	*P* value
Age (years)	1.322 (0.962–1.818)	0.085
Female/male	6.164 (0.249–152.841)	0.267
BMI (kg/m^2^)	1.973 (0.996–3.909)	0.051
ASA (scores)	3.286 (0.486–22.224)	0.223
Preoperative comorbidities		
Diabetes mellitus	7.629 (0.149–13.125)	0.312
Hypertension	32.862 (0.358–313.601)	0.130
Coronary artery disease	0.008 (0.000–14.263)	0.206
Peripheral vascular disease	19.901 (0.663–58.988)	0.069
Central nervous system disease	5.294 (1.601–9.850)	0.038
Hemoglobin (g/L)	0.927 (0.845–1.016)	0.105
Platelets × 10^9^/L	0.980 (0.933–1.030)	0.428
White blood cells × 10^9^/L	0.522 (0.103–2.969)	0.489
Lymphocyte × 10^9^/L	1.730 (0.639–4.285)	0.090
CRP (mg/L)	0.424 (0.132–1.365)	0.150
Alb (g/L)	1.196 (0.792–1.806)	0.395
CAR	1.955 (0.000–1.255)	0.143
NLR	2.194 (0.160–3.136)	0.557
PLR	1.005 (0.995–1.057)	0.856
The operation time (min)	3.105 (1.544–6.247)	0.001

**Aberrative:** POD, postoperative delirium; OR, Odds ratio; CI, Confidence interval; CRP, C-reactive protein; Alb, albumin; CAR, C-reactive protein to albumin Ratio; NLR, neutrophil-lymphocyte ratio; PLR, platelet-lymphocyte ratio; min, minutes. *P* < 0.05 was considered statistically significant.

## Discussion

The present study was designed to assess the correlations of CRP, Alb and CAR in patients with POD. Our results showed that CRP and CAR were higher in POD patients than those in patients without POD. In addition, we identified a significantly decreased Alb in POD patients. Moreover, the MADS score used to assess POD activity showed a strong correlation with CAR. Therefore, we demonstrated that CAR may be significant predictors for disease activity in POD patients. The results also showed that age, ASA scores and the operation time was an independent predictor for patients with POD.

There is growing evidence has indicated that POD and inflammation are linked. However, the specific mechanism between POD and inflammatory response is still unclear, and the possible reasons are as follows: (1) the dysregulation of cytokines, including free radicals, complement factors, glutamate, and nitric oxide, caused by the activation of a systemic inflammatory cascade, is believed to be a key cause of neurodegeneration and subsequent cognitive impairment, which in turn impair cognitive ability (24). (2) the activation of the inflammatory cascade alters peripheral and central cytokines, leading to degenerative inflammatory processes in the central nervous system that clinically manifest as cognitive impairment when the cognitive reserve is destroyed (25).

Among the various markers of inflammation, CRP is a reactant in the acute phase and is often used as a monitoring biomarker for inflammatory response (26). Previous studies have shown that delirium and sepsis in patients after hip surgery are also associated with elevated CRP levels (27). Alb is a commonly used parameter to evaluate the nutritional status of surgical patients. Studies have shown that hypoalbuminemia is significantly associated with an increased risk of postoperative complications, including POD (28). However, the predictive effect of CAR on POD has rarely been reported. Therefore, we evaluated the value of the CAR as a valid predictor of POD after TKA in patients with old than 60 years. Currently, there are few reports on the relationship between CAR and POD. Most studies have focused on the relationship between CRP and POD (29, 30). At the same time, several clinical studies have investigated the relationship between CRP and POD in elderly patients (30), and they found that CRP levels were highly correlated with the onset of delirium. Macdonald et al. (31) even suggested that CRP could predict the occurrence and recovery of delirium. The results of this study are consistent with previous studies, our study further found that patients with high CAR had a higher risk of POD after surgery. In addition, we used multiple regression analysis after adjusting for confounders to clearly show that CAR and ASA scores are independent risk factors for postoperative delirium

Previous studies have shown that postoperative adverse outcomes are closely related to systemic inflammatory responses following surgical stress, characterized by increased CRP and decreased Alb level (27, 32). The CAR, a combination of two circulating acute phase proteins, is an inflammation-based prognostic factor that can be used as a prognostic factor in postoperative patients (33). According to a recent report, the CAR can help predict the risk of postoperative death in elderly patients with hip fracture (34), and the combination of CRP and Alb with POD was more likely to reflect preexisting inflammatory stress than CRP or Alb alone. There is compelling evidence that the release of proinflammatory cytokines into the peripheral and central nervous system plays a crucial role in the pathogenesis of POD (7–9). Therefore, the pathogenic role of systemic inflammation in POD may be a possible explanation for the predictive effect of CAR on POD in this study. In our study, it was also found that the AUC value of CRP/Alb in the diagnosis of POD was 0.782, accompanied by a good 83.3% sensitivity. The value of CRP/Alb in the diagnosis of POD after surgery was significantly higher than that of CRP and Alb, providing an effective predictor for clinical prediction of postoperative delirium.

Due to the complex causes of delirium, there is no consensus on the risk factors of postoperative delirium in elderly patients with total knee arthroplasty, and there is no effective predictive method to assess the risk of delirium. In this study, the preoperative clinical data, blood routine and biochemical indexes of 268 patients over 60 years old undergoing TKA were compared, and it was found that there were statistical differences in CRP, Alb, CAR between the delirium group and the without delirium. We further introduced the multivariate binary regression analysis, and found that age, ASA scores and the operation time were also the risk factors for perioperative delirium in TKA patients older than 60 years, which indicated that perioperative delirium was the result of the joint influence of multiple factors.

CRP/Alb as a predictor of perioperative delirium in elderly patients with TKA has certain rationality and feasibility. First, the index is consistent with the inflammatory theory of delirium (7). With the increase of age, the compensatory function of the body in elderly patients decreases significantly. TKA is mostly found in elderly patients older than 60 years old, and since the trauma, surgery and secondary infection, and inflammatory response will occur, but the nutritional status will decline. In this study, the mean CRP of patients in the delirium group was 20.33 mg/L, which was about 2 times higher than the normal range. The mean albumin was 39.16 g/L, lower than the normal range. Previous studies have shown that the release of inflammation will result in increased dopamine, activation of endothelial tissue and subsequent destruction of blood-brain barrier, nervous system ischemia and eventually delirium. Second, the results are clinically feasible. CRP is a nonspecific indicator of acute inflammation in the body and represents the state of the immune system (27). The serum Alb level, as an evaluation index of protein and energy consumption, represents the body's systemic nutritional status (28). At present, the detection methods of CRP and Alb are mature and have good uniformity and standardization. CRP/Alb, as the ratio of the two, is simple and easy to calculate, and has been widely used as a clinical reference to evaluate the prognosis of various surgical systems.

More importantly, it is important to note that the care plan is rich in non-pharmacological measures for delirium management, and studies have shown that the use of non-pharmacological treatment with polygenic strategies can reduce the incidence of delirium in patients (35), thereby reducing the cost to the health care establishment. Therefore, by predicting the incidence of delirium preoperatively, making a good nursing plan, strengthening perioperative nursing management as early as possible, realizing early treatment of delirium, ultimately reducing medical costs and improving the purpose of rapid recovery of patients (36).

This study still has some limitations. First of all, this study was a retrospective, single-center study with limited cases included, and there was a certain degree of selective bias. Secondly, the factors affecting POD and MADS are diverse, and there are other factors besides inflammation, such as age, ASA scores, and operation of surgery, but the remaining confounders may affect POD, but how these confounders affect POD remains unclear and requires further investigation in the future. In addition, this study did not consider the change of delirium in the pathogenesis of intracranial factor, such as serotonin, and acetylcholine, jointly determine the risk and prognosis of delirium. Third, given the insufficient sample size, further internal and external validation is needed in the future for the validity of CAR to determine the feasibility of POD.

## Conclusion

To sum up, we have demonstrated that the CAR is an independent risk factor for and predicts postoperative delirium in patients older than 60 years of age undergoing TKA, which provides potential recommendations for early intervention in delirium care.

## Data Availability

The raw data supporting the conclusions of this article will be made available by the authors, without undue reservation.

## References

[1] Dalury DF. Cementless total knee arthroplasty: Current concepts review. Bone Joint J. (2016) 98-B(7):867–73. 10.1302/0301-620X.98B7.37367 27365463

[2] Sorel JC Veltman ES Honig A Poolman RW. The influence of preoperative psychological distress on pain and function after total knee arthroplasty: A systematic review and meta-analysis. Bone Joint J. (2019) 101-B(1):7–14. 10.1302/0301-620X.101B1.BJJ-2018-0672.R1 30601044

[3] Boyce L Prasad A Barrett M Dawson-Bowling S Millington S Hanna SA The outcomes of total knee arthroplasty in morbidly obese patients: A systematic review of the literature. Arch Orthop Trauma Surg. (2019) 139(4):553–60. 10.1007/s00402-019-03127-5 30778723 PMC6420900

[4] Jin Z Hu J Ma D. Postoperative delirium: Perioperative assessment, risk reduction, and management. Br J Anaesth. (2020) 125(4):492–504. 10.1016/j.bja.2020.06.063 32798069

[5] Janssen TL Alberts AR Hooft L Mattace-Raso F Mosk CA van der Laan L. Prevention of postoperative delirium in elderly patients planned for elective surgery: Systematic review and meta-analysis. Clin Interv Aging. (2019) 14:1095–117. 10.2147/CIA.S201323 31354253 PMC6590846

[6] Hughes CG Boncyk CS Culley DJ Fleisher LA Leung JM McDonagh DL American Society for enhanced recovery and perioperative quality initiative joint consensus statement on postoperative delirium prevention. Anesth Analg. (2020 Jun) 130(6):1572–90. 10.1213/ANE.0000000000004641 32022748 PMC7379173

[7] Rudolph JL Marcantonio ER. Review articles: Postoperative delirium: Acute change with long-term implications. Anesth Analg. (2011) 112(5):1202–11. 10.1213/ANE.0b013e3182147f6d 21474660 PMC3090222

[8] Evered LA Silbert BS. Postoperative cognitive dysfunction and noncardiac surgery. Anesth Analg. (2018) 127(2):496–505. 10.1213/ANE.0000000000003514 29889707

[9] Fong TG Davis D Growdon ME Albuquerque A Inouye SK. The interface between delirium and dementia in elderly adults. Lancet Neurol. (2015) 14(8):823–32; Erratum in: *Lancet Neurol*. (2015) **14**(8):788. 10.1016/S1474-4422(15)00101-5 26139023 PMC4535349

[10] Bélanger L Ducharme F. Patients’ and nurses’ experiences of delirium: A review of qualitative studies. Nurs Crit Care. (2011) 16(6):303–15. 10.1111/j.1478-5153.2011.00454.x 21999421

[11] Simone MJ Tan ZS. The role of inflammation in the pathogenesis of delirium and dementia in older adults: A review. CNS Neurosci Ther. (2011) 17(5):506–13. 10.1111/j.1755-5949.2010.00173.x 20553303 PMC6493838

[12] Schreuder L Eggen BJ Biber K Schoemaker RG Laman JD de Rooij SE. Pathophysiological and behavioral effects of systemic inflammation in aged and diseased rodents with relevance to delirium: A systematic review. Brain Behav Immun. (2017) 62:362–81. 10.1016/j.bbi.2017.01.010 28088641

[13] Subramaniyan S Terrando N. Neuroinflammation and perioperative neurocognitive disorders. Anesth Analg. (2019) 128(4):781–8. 10.1213/ANE.0000000000004053 30883423 PMC6437083

[14] Varatharaj A Galea I. The blood-brain barrier in systemic inflammation. Brain Behav Immun. (2017) 60:1–12. 10.1016/j.bbi.2016.03.010 26995317

[15] Pol RA van Leeuwen BL Izaks GJ Reijnen MM Visser L Tielliu IF C-reactive protein predicts postoperative delirium following vascular surgery. Ann Vasc Surg. (2014) 28(8):1923–30. 10.1016/j.avsg.2014.07.004 25017770

[16] Slor CJ Witlox J Adamis D Jansen RWMM Houdijk APJ van Gool WA The trajectory of C-reactive protein serum levels in older hip fracture patients with postoperative delirium. Int J Geriatr Psychiatry. (2019) 34(10):1438–46. 10.1002/gps.5139 31058343

[17] Artigas A Wernerman J Arroyo V Vincent JL Levy M. Role of albumin in diseases associated with severe systemic inflammation: Pathophysiologic and clinical evidence in sepsis and in decompensated cirrhosis. J Crit Care. (2016) 33:62–70. 10.1016/j.jcrc.2015.12.019 26831575

[18] Cabrerizo S Cuadras D Gomez-Busto F Artaza-Artabe I Marín-Ciancas F Malafarina V. Serum albumin and health in older people: Review and meta analysis. Maturitas. (2015) 81(1):17–27. 10.1016/j.maturitas.2015.02.009 25782627

[19] Avan A Tavakoly Sany SB Ghayour-Mobarhan M Rahimi HR Tajfard M Ferns G. Serum C-reactive protein in the prediction of cardiovascular diseases: Overview of the latest clinical studie s and public health practice. J Cell Physiol. (2018) 233(11):8508–25. 10.1002/jcp.26791 29932219

[20] Shen Y Wang H Li W Chen J. Prognostic significance of the CRP/alb and neutrophil to lymphocyte ratios in hepatocellular carcinoma patients undergoing TACE and RFA. J Clin Lab Anal. (2019) 33(9):e22999. 10.1002/jcla.22999 31418936 PMC6868405

[21] Hu H Yao X Xie X Wu X Zheng C Xia W Prognostic value of preoperative NLR, dNLR, PLR and CRP in surgical renal cell carcinoma patients. World J Urol. (2017) 35(2):261–70. 10.1007/s00345-016-1864-9 27255479

[22] Katzman R Zhang MY Ouang-Ya-Qu WZ Liu WT Yu E Wong SC A Chinese version of the Mini-mental state examination; impact of illiteracy in a Shanghai dementia survey. J Clin Epidemiol. (1988) 41(10):971–8. 10.1016/0895-4356(88)90034-0 3193141

[23] Inouye SK Westendorp RG Saczynski JS. Delirium in elderly people. Lancet. (2014) 383(9920):911–22. 10.1016/S0140-6736(13)60688-1 23992774 PMC4120864

[24] Steiner LA. Postoperative delirium. Part 1: Pathophysiology and risk factors. Eur J Anaesthesiol. (2011) 28(9):628–36. 10.1097/EJA.0b013e328349b7f5 21785356

[25] Kalvas LB Monroe TB. Structural brain changes in delirium: An integrative review. Biol Res Nurs. (2019) 21(4):355–65. 10.1177/1099800419849489 31067980 PMC6794667

[26] Del Giudice M Gangestad SW. Rethinking IL-6 and CRP: Why they are more than inflammatory biomarkers, and why it matters. Brain Behav Immun. (2018) 70:61–75. 10.1016/j.bbi.2018.02.013 29499302

[27] Cerejeira J Nogueira V Luís P Vaz-Serra A Mukaetova-Ladinska EB. The cholinergic system and inflammation: Common pathways in delirium pathophysiology. J Am Geriatr Soc. (2012) 60(4):669–75. 10.1111/j.1532-5415.2011.03883.x 22316182

[28] Öztürk Birge A Bedük T. The relationship of delirium and risk factors for cardiology intensive care unit patients with the nursing workload. J Clin Nurs. (2018) 27(9-10):2109–19. 10.1111/jocn.14365 29603815

[29] Egberts A Osse RJ Fekkes D Tulen JHM van der Cammen TJM Mattace-Raso FUS. Differences in potential biomarkers of delirium between acutely ill medical and elective cardiac surgery patients. Clin Interv Aging. (2019) 14:271–81. 10.2147/CIA.S193605 30799917 PMC6369845

[30] Dunne SS Coffey JC Konje S Gasior S Clancy CC Gulati G Biomarkers in delirium: A systematic review. J Psychosom Res. (2021) 147:110530. 10.1016/j.jpsychores.2021.110530 34098376

[31] Macdonald A Adamis D Treloar A Martin F. C-reactive protein levels predict the incidence of delirium and recovery from it. Age Ageing. (2007) 36(2):222–5. 10.1093/ageing/afl121 17114198

[32] Chi YL Zhang WL Yang F Su F Zhou YK. Transcutaneous electrical acupoint stimulation for improving postoperative recovery, reducing stress and inflammatory responses in elderly patient undergoing knee surgery. Am J Chin Med. (2019) 47(7):1445–58. 10.1142/S0192415X19500745 31752522

[33] Liu Z Jin K Guo M Long J Liu L Liu C Prognostic value of the CRP/alb ratio, a novel inflammation-based score in pancreatic cancer. Ann Surg Oncol. (2017) 24(2):561–8. 10.1245/s10434-016-5579-3 27650825

[34] Chen L Zhang J Zhang W Deng C. Correlation between C-reactive protein/albumin and contralateral hip refracture after total hip arthroplasty in elderly patients with hip fractures. Ann Palliat Med. (2020) 9(3):1055–61. 10.21037/apm-20-855 32434368

[35] Pérez-Ros P Martínez-Arnau FM Baixauli-Alacreu S Caballero-Pérez M García-Gollarte JF Tarazona-Santabalbina F. Delirium predisposing and triggering factors in nursing home residents: A cohort trial-nested case-control study. J Alzheimers Dis. (2019) 70(4):1113–22. 10.3233/JAD-190391 31322572

[36] Piao J Jin Y Lee SM. Triggers and nursing influences on delirium in intensive care units. Nurs Crit Care. (2018) 23(1):8–15. 10.1111/nicc.12250 27353862

